# Seasonality of sightings of *Cacajao ucayalii* at the Estación Biológica Quebrada Blanco and implications concerning ranging patterns and habitat use

**DOI:** 10.5194/pb-12-9-2025

**Published:** 2025-09-15

**Authors:** Eckhard W. Heymann, Camilo Flores Amasifuén, Ney Shahuano Tello

**Affiliations:** 1 Verhaltensökologie & Soziobiologie, Deutsches Primatenzentrum – Leibniz-Institut für Primatenforschung, 37077 Göttingen, Germany; 2 Estación Biológica Quebrada Blanco, Río Tahuayo, Loreto, Peru; 3 Asociación Científica y de Turismo Comunitario Quebrada Blanco, Tahuantinsuyo 436, Iquitos, Loreto, Peru

## Abstract

This paper reports long-term data on the seasonality of sightings of Ucayali bald uakaris, *Cacajao ucayalii* at the Estación Biológica Quebrada Blanco (EBQB). Sightings were most common during the drier parts of the year and rarer during the rainy season. This suggests that the *C. ucayalii* groups travel long distances and seasonally move (migrate) between different parts of presumably large home ranges and between different habitats (*terra firme* forest, Amazon floodplain forest). Areas to protect this Neotropical primate must be large enough to allow for sufficient seasonal access to these vegetation types.

## 1 Introduction

Most tropical rainforests – home to the majority of primate species (Estrada et al., 2017) – have seasonal patterns of plant phenology, i.e. leaf flushing, flowering, and fruiting (van Schaik et al., 1993). This results in seasonal variation in the availability of food resources for primary consumers. All primates, except tarsiers, include plant material in their diet, and, for the majority, fruit is the primary food resource (Harding, 1981; Richard, 1985). Primates may respond to this variation through dietary changes, e.g. by increasing or decreasing their dietary diversity, by switching food types, and/or by changing their ranging patterns through increasing or decreasing home-range size and daily travel path lengths (Hemingway and Bynum, 2005). Primates may also temporarily switch habitat types, i.e. move from one habitat with reduced food availability to another habitat that is phenologically out of synchrony with the former (van Schaik and Brockman, 2005).

In Neotropical rainforests, although fruit production can be aseasonal, it generally peaks more often in the rainy season than at other times of the year (Mendoza et al., 2017, 2018). More specifically, the peak of ripe fruits largely falls into the rainy season, while the peak of unripe fruits precedes the rainy season (Peres, 1994).

The Ucayali bald uakari, *Cacajao ucayalii* (previously named *Cacajao calvus rubicundus* and *Cacajao calvus ucayalii*; see Silva et al., 2022), is a medium-sized (2.3–3.5 kg) primate that feeds mainly on seeds (Bowler and Bodmer, 2011). They live in groups of up to 200 individuals that fission into smaller units (Aquino, 1988, 1998; Bowler et al., 2012). Based on a short survey in the Río Tapiche area in north-eastern Peru, Fontaine (1979, 1981) considered *C. ucayalii* to be specialized for living in flooded forests (see Barnett et al., 2013, for a general overview of the genus). However, an analysis of published sightings revealed that these uakaris are flexible in their habitat requirements and generally use *terra firme* forests or a mixture of habitats (Heymann and Aquino, 2010). Earlier, Heymann (1992) and Aquino (1998) had suggested that *C. ucayalii* may undertake seasonal migrations. Bowler (2007), in the only detailed ecological study of *C. ucayalii*, on the Río Yavarí, found a clear seasonal pattern in the use of three different habitats (*terra firme*, *Mauritia flexuosa* swamps, and flooded forest). He also noted that “uakari groups regularly left the study area and … could not be located until they re-entered the area” (p. 124). Thus, the 12 km^2^ estimate for the home-range size is likely to represent a minimum estimate (Bowler, 2007). In line with this, Leonard and Bennett (1996) estimated a home-range size of more than 30 km^2^.

Since 1985, field researchers at the Estación Biológica Quebrada Blanco (EBQB) in north-eastern Peru have recorded *C. ucayalii* moving through, feeding, and sleeping in the study area (Bartecki and Heymann, 1987; Heymann, 1990). Attempts to follow the uakaris beyond the 1 km^2^ study area failed, due to the impossibility of keeping up with the fast-moving animals in difficult terrain without an extensive trail system. The intermittent sightings of *C. ucayalii* indicated that the EBQB study area represented only a fraction of their home-range area and that they pass through the area while moving between wider areas and perhaps between different habitats. In order to examine whether this could support the notion of seasonal migrations quoted above, we compiled all information on sightings of *C. ucayalii* at EBQB and analysed their temporal distribution.

## 2 Methods

The data on which this paper is based stem from observations at the Estación Biológica Quebrada Blanco (EBQB), a field research site established in 1984 by the Proyecto Peruano de Primatología (Iquitos, Peru) and run by the Deutsches Primatenzentrum (DPZ) from 1997 to 2022. EBQB is located at 4°21^′^ S, 73°09^′^ W in a largely intact lowland tropical rainforest of north-eastern Peruvian Amazonia. The habitat in the EBQB area is characterized by primary *terra firme* forest (“*bosque de altura*” in the terminology of Encarnación, 1985) on flat to strongly undulating terrain (“*bosque de terraza*” and “*bosque de colina*”; Encarnación, 1985), interspersed with small swampy areas along creeks. Annual rainfall averages around 3000 mm, with December to May receiving on average > 250 mm and July to September < 200 mm (data from the nearest meteorological station at Tamshiyacu, 40 km north of EBQB). For further details of EBQB, see Heymann and Tirado Herrera (2021) and Heymann et al. (2021).

Local field assistants, students, and researchers working at EBQB were requested to report sightings of *C. ucayalii*, including information on date, time of day, location within the EBQB study area, group size, feeding records, and association with other primate species. This resulted in 56 sightings between 1985 and 2022. Local field assistants, particularly Camilo Flores Amasifuén and Ney Shahuano Tello, who were present at EBQB throughout most of the year, reported the majority of sightings.

During behavioural research on tamarins and titi monkeys, we made monthly phenological records (restricted to plant species exploited by tamarins and titi monkeys) in 1997–1998, 2000–2001, and 2005. Following Peres (1994) we scored ripe fruits as present or absent and calculated monthly fruiting indices as the number of trees with ripe fruits present divided by the number of trees examined ( n = 216). For statistical analyses, we lumped sightings, rainfall, and fruit phenology scores into 2-month bins (January–February, March–April, etc.). We calculated the Spearman rank order correlation between the number of bimonthly sightings and between bimonthly rainfall and ripe-fruit phenology scores, respectively, in Statistica v.14 (TIBCO, 2020).

The data for this paper were collected during research authorized by different Peruvian authorities (Ministerio de Agricultura, Lima; Dirección Regional de Recursos Naturales, Iquitos; Instituto Nacional de Recursos Naturales, INRENA; Servicio Nacional Forestal y de Fauna Silvestre, SERFOR) since 1985.

## 3 Results and discussion

**Figure 1:**
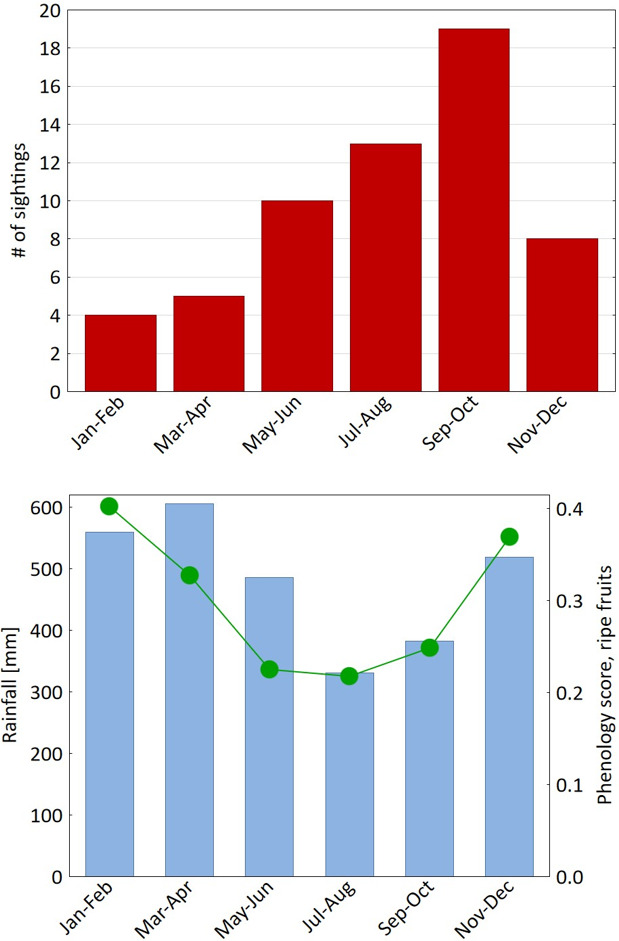
**(a)** Temporal (bimonthly) distribution of sightings (bars) of *Cacajao ucayalii* at EBQB. **(b)** Rainfall (blue bars) and ripe-fruit phenology scores (green dots).

The distribution of sightings of *C. ucayalii* at EBQB is uneven across the year: sightings peak in September–October and show a minimum in the first 4 months of the year (Fig. 1). The majority of sightings took place between the final part of the rainy season (May) and the late dry season (October) (for long-term monthly rainfall patterns, see Lüffe et al., 2018). The distribution of sightings is significantly negatively correlated with rainfall (Spearman r = - 0.89, p < 0.05) and negatively but not significantly correlated with the ripe-fruit phenology scores (Spearman r = - 0.77, n.s.).

**Figure 2:**
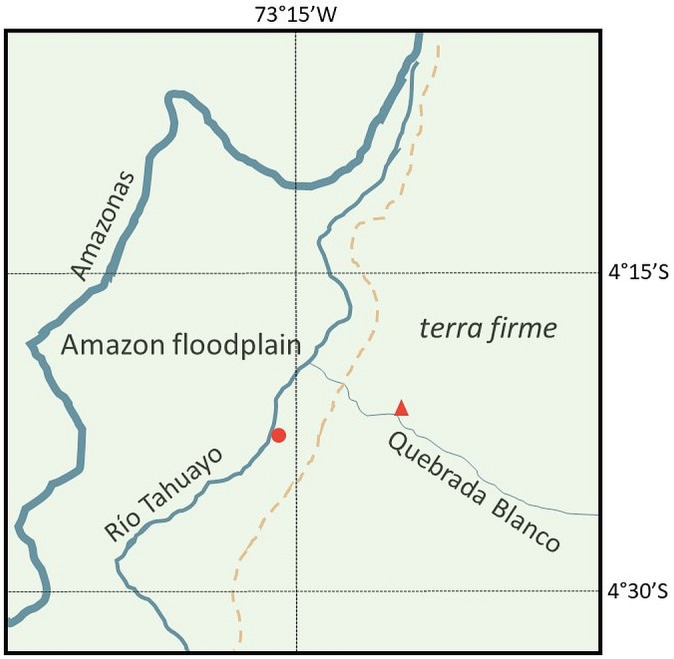
Location of EBQB (red triangles) in the *terra firme* and ARC (red dots) in the Amazon floodplain. The dashed line indicates the border between *terra firme* and floodplain. Drawn from the “Mapa geoecológico de la zona de Iquitos” in Kalliola and Flores Paitán (1998).

The temporal distribution of sightings supports the notion of seasonal movements or migrations between different areas of the home range and/or different habitats. EBQB is located on a *terra firme* terrace above the Amazon floodplain. We suspect that groups of *C. ucayalii* move between the *terra firme* and the Amazon floodplain, which extends to the lower Quebrada Blanco and to the Río Tahuayo. This is supported by information on sightings of *C. ucayalii* in the study area of the Amazon Research Center (ARC) on the Río Tahuayo, above the confluence with Quebrada Blanco, an area located within the Amazon floodplain (Fig. 2). During a study on *Pithecia monachus* at ARC between July 2019 and June 2020 (Gottstein et al., 2023), *C. ucayalii* was seen in the study area in March, April, and May 2020 when the forest was flooded, but not during other parts of the year (Malika Gottstein, personal communication, 2025). Similarly, Larissa Barker (personal communication, 2025) and Nick Gardner (personal communication, 2025) have encountered *C. ucayalii* (probably two distinct groups) more reliably in the ARC area in the rainy season during their respective primatological and ornithological research. One of the two groups seemed to frequent an *aguajal* (*Mauritia flexuosa* swamp) in the hinterland of the ARC area, while the other seemed to stay closer to the Río Tahuayo.

The straight-line distance from EBQB to this floodplain forest is ca. 6 km to the ARC area ca. 12 km (Fig. 2). Bowler (2007) and Leonard and Bennett (1996) reported daily travel path lengths of 6 and 7.3 km, respectively, for *C. ucayalii*. Thus, they could easily travel from the EBQB and areas further up Quebrada Blanco to the floodplain forest within a few days. Although rare amongst primates, habitat shifting associated with travelling over larger distances has been reported for some species (see summary in Knott and DiGiorgio, 2024).

Seasonal variation in the availability of food is a possible factor influencing long-distance travelling in primates. According to Haugaasen and Peres (2005) fruit availability differs between *terra firme* and floodplain forest: in the former, fruiting peaks at the onset of the rainy season; in the latter it is concentrated during the inundation period. At EBQB, ripe-fruit availability peaks in December and January, i.e. in the early rainy season (Lüffe et al., 2018). Since uakaris are mainly predators of both unripe and mature seeds (Bowler and Bodmer, 2011), we would not expect that sightings coincide with the availability of ripe fruits but rather with the availability of unripe fruits. Phenology records in Brazilian and Ecuadorian Amazonia indicate that peaks in the availability unripe fruit precede peaks in the availability of ripe fruits by 4 months (Peres, 1994; DiFiore, 2004). If we shift the phenology scores by 4 months, the peak in sightings coincides with the peak in unripe-fruit (and thus unripe seed) availability at EBQB.

We admit that our scenario is hypothetical. It can only be tested by following groups of *C. ucayalii* over long times and large distances, which is technically very challenging. Attempts to put radio collars on *C. ucayalii* failed (Suzie Leonard, personal communication, 1995). A possible solution would be the use of drones: wider areas could be surveyed for groups of *C. ucayalii*, and, once encountered, groups could be followed over larger distances.

The question of whether or not *C. ucayalii* undertakes large-scale movements between habitats is not only of scientific interest but also relevant for their conservation. Such long distances travelled by these uakari groups indicate that only areas large enough to allow for such movements can sustainably protect them. The Área de Conservación Regional-Comunal Tamshiyacu-Tahuayo (ACRCTT) is such an area, as it harbours still largely intact forest, despite being located close to the major Amazonian city of Iquitos.

## Data Availability

The dataset on which this paper is based is available at 10.5281/zenodo.16994344 (Heymann et al., 2025).

## Code availability

As stated in the Methods section, data were analysed with Statistica v.14 (http://tibco.com, TIBCO, 2020). No specific code was programmed.
